# On the Use of Oxy-Chloride of Zinc over Exposed Pulps

**Published:** 1868-02

**Authors:** I. A. Salmon


### ON THE USE OF OXY-CHLORIDE OF ZINC OVER EPXOSED PULPS.

Read before the Massachusetts Dental Association, May, 1867.

BY I. A. SALMON.

At a former meeting of this Society I took occasion to advocate the use of oxy-chloride of zinc over exposed pulps, as suggested to me by Dr. Keep, and at that time read to the society the result of a few cases occurring in my practice treated in this manner. The result to that time having been so favorable, I have since used it with a great degree of confidence. Could our brothers of the profession be induced to give it a fair trial, I feel sure its use would be very generally adopted, and the present various modes of capping, so often necessitating the use of temporary fillings, and so uncertain in their results, would be dispensed with.

To use oxy chloride of zinc successfully, considerable care must be exercised. It is important that the materials be pure and properly prepared.

The oxide of zinc is often impure, containing white lead, chalk and other substances, that of a white color is not considered of as good quality as the yellowish white.

Should there be an excess of the chloride of zinc, its escharotic property will be strongly marked. The strength of the solution used should be only sufficient to cause the mixture to set.

My method of manipulation is to cut from fine linen a small piece sufficient to cover that part of the pulp I desire to protect; having mixed the oxy-chloride, the piece of linen is saturated with it, a portion being applied to one or both sides, which is then carried upon an instrument and placed directly over the point desired to protect. More or less pain is occasioned which, however, speedily subsides and does not return.

After a few minutes and as soon as the mixture is firmly set, during which time moisture must be excluded from the cavity, I introduce the gold and proceed as in ordinary cases.

I have kept a record of most of the cases in which I have used the oxy-chloride of zinc and have arranged them in the following tabular order; as facts cannot be disputed, I will give it:

| Why used. | No. of Cases. | When permanently filled. |
|---|---|---|
| To protect pulps (not exposed) | 44 | At the same sitting. |
| Over exposed pulps | 27 | 21 at the same sitting. |
| | | 1 in about one week. |
| | | 2 " two " |
| | | 1 " three " |
| | | 1 " four " |
| | | 1 " eight " |
| Over exposed & bleeding pulps, | 7 | 1 at the same sitting. |
| | | 1 in about one week. |
| | | 3 " two " |
| | | 1 " three " |
| | | 1 " four " |
| Making a total of | 78 | cases, in thirty-four (34) of which the pulp was exposed. |

In every case which I have subsequently examined, I have found the tooth perfectly healthy and apparently as sensitive as before the application, and as far as I am aware have not had a failure.

