# Correction

**Published:** 1868-04

**Authors:** 


### CORRECTION.

The communication in the February No. of the REGISTER, page 69, on "The use of Oxy-Cloride of Zinc over exposed Pulps," bears the name of Dr. I. A. Salmon. Though the Dr. endorses the article, he does not wish to receive the credit that belongs to another. The article was written by Dr. T. B. Hitchcock. The paper, when it passed into the hands of the printer, had not the writer's name attached; inadvertantly Dr. Salmon's name was affixed. We will again express what we have so often suggested, viz.: That copy should be prepared as perfectly as the writer is capable of doing it, leaving nothing omitted that he desires to appear. Often communications come to us without a caption, without a signature, and sometimes with very few punctuation marks. When anything is to be said it should be well said, and plainly said. Illigibility in chirography is a common and very sad fault of writers, and one, too, for which there is very little apology, except slovenly carelessness. We do not mean to say that these remarks apply to Dr. H.'s paper. T.

